# Chikungunya clusters in the state of Bahia: influence of environmental and social factors

**DOI:** 10.1590/0037-8682-0321-2025

**Published:** 2025-12-12

**Authors:** Maryly Weyll Sant´Anna, Raquel Gardini Sanches Palasio, Alec Brian Lacerda, Maurício Lamano Ferreira, Francisco Chiaravalloti-Neto, Fabricio Bau Dalmas, Pedro Luiz Côrtes

**Affiliations:** 1 Universidade de São Paulo, Instituto de Energia e Ambiente, São Paulo, SP, Brasil. Universidade de São Paulo Instituto de Energia e Ambiente São Paulo SP Brasil; 2 Universidade de São Paulo, Faculdade de Saúde Pública, Laboratório de Análise Espacial em Saúde, Departamento de Epidemiologia, São Paulo, SP, Brasil. Universidade de São Paulo Faculdade de Saúde Pública Laboratório de Análise Espacial em Saúde São Paulo SP Brasil; 3 Universidade de São Paulo, Escola de Engenharia de Lorena, Departamento de Ciências Básicas e Ambientais, Lorena, SP, Brasil. Universidade de São Paulo Escola de Engenharia de Lorena Departamento de Ciências Básicas e Ambientais Lorena SP Brasil; 4 Universidade de Guarulhos, Programa de Mestrado em Análise Ambiental, Guarulhos, SP, Brasil. Universidade de Guarulhos Programa de Mestrado em Análise Ambiental Guarulhos SP Brasil; 5 Universidade de São Paulo, Escola de Comunicações e Artes, São Paulo, SP, Brasil. Universidade de São Paulo Escola de Comunicações e Artes São Paulo SP Brasil

**Keywords:** Arbovirus, Chikungunya, Social determinants, Environmental factors, Spatial analysis

## Abstract

**Background:**

Chikungunya is an emerging disease that significantly impacts global public health and is associated with various environmental and social factors. This study aimed to identify the spatial and spatiotemporal clusters of chikungunya in the state of Brazilian Bahia, as well as their relationships with environmental and socioeconomic variables.

**Methods:**

High- and low-risk clusters were analyzed for 2014-2023 using SatScan. Associations among socioeconomic, climatic, and vegetation characteristics were established using geostatistical estimates.

**Results:**

Many high-risk clusters were observed at high densities in the southern, north-central, and south-central mesoregions. The months with the highest risk were February and March. A decreasing chikungunya trend of −0.6% per year was identified in the Bahian territory when the spatial variation of the temporal trends was analyzed. High-risk municipalities within the spatial chikungunya clusters generally had higher minimum annual and summer temperatures, lower thermal amplitudes, higher monthly and average summer precipitation levels, and higher socioeconomic indicators. The lowest vegetation cover was observed in the Caatinga biome, and the highest in the Atlantic Forest.

**Conclusions:**

Bahia has many high-risk clusters for chikungunya, underscoring the need to strengthen preventive and control measures through coordinated epidemiological surveillance services across the state.

## INTRODUCTION

Chikungunya virus (CHIKV) has emerged as a global public health threat with high pandemic potential, spreading to new geographic regions, including Europe and North America^1^^,^^2^. Chikungunya (CHIK) is an infectious disease transmitted to humans by Aedes spp (Meigen, 1818) mosquitoes infected with CHIKV^3^. In Brazil, the main vector of this disease is Aedes aegypti (Linnaeus, 1762); however, Ae. Albopictus (Skuse, 1894) is also a competent vector present throughout Brazil^4^. Climatic factors (temperature increase), unplanned urban growth, deforestation, and inadequate urban infrastructure, such as poor sanitation and waste management, contribute to CHIK outbreaks^5^. These conditions increase the number of artificial breeding sites, alter vector behavior, and increase vector-host interactions, thereby increasing the risk of infection^6^^,^^7^.

CHIK symptoms include fever, rash, headache, and severe joint pain, often overlapping with those of dengue and Zika^8^. Although most patients progress benignly, some progress to chronic and severe forms, with complications involving the central nervous system, liver dysfunction, and even death^9^^,^^10^. Studies in Puerto Rico^11^ and Brazil^12^^-^^14^ have reported associations between CHIKV infection and increased mortality. Further, deaths have been linked to neurological, endocrine, and cardiac disorders, possibly owing to inflammatory tissue damage during the viremic period^14^. As deaths generally occur between 30 and 85 days post-infection, this association becomes more difficult and contributes to the underreporting of CHIKV-associated mortality^12^^,^^14^. A possible explanation is the ability of CHIKV to persist in musculoskeletal cells involved in tissue regeneration^15^. Consequently, this arbovirus remains a major global public health challenge, especially in the context of climate change intensified by urbanization^16^. 

CHIK was first recorded in the Americas in late 2013 in the Caribbean and reached Brazil in July 2014, with the first autochthonous case confirmed in August in Amapá^4^. In September 2014, an outbreak occurred in Feira de Santana, Bahia, reported by the Brazilian Ministry of Health. Since then, recurrent and intense outbreaks have spread across Brazil, especially in the Northeast, with Bahia being among the states with the highest number of cases and showing an increasing trend in both intensity and geographic distribution^5^.

Given its large population, ecological diversity, and history of CHIK outbreaks, Bahia is a critical area for epidemiological surveillance, being among the Brazilian states with the highest number of CHIK cases and historical outbreaks, with an increase in outbreak intensity and expansion in recent years^17^^,^^18^. Although a few studies^19^^-^^22^ have analyzed municipalities such as Salvador and Feira de Santana, most remain restricted to neighborhood-level assessments. Studies in Bahia have linked CHIK transmission to local environmental and socioeconomic conditions, including uncovered water storage containers, vegetation cover, and temperature variations^21^^,^^22^. However, spatial studies on CHIK transmission in the state remain limited. Thus, this study aimed to identify spatiotemporal clusters of high risk for CHIK in Bahia, and to examine the influence of climatic and social factors. The approach used in this study may support improved disease control actions and strengthen prophylactic strategies in high-risk areas.

## METHODS

### Study area

**FIGURE 1:**
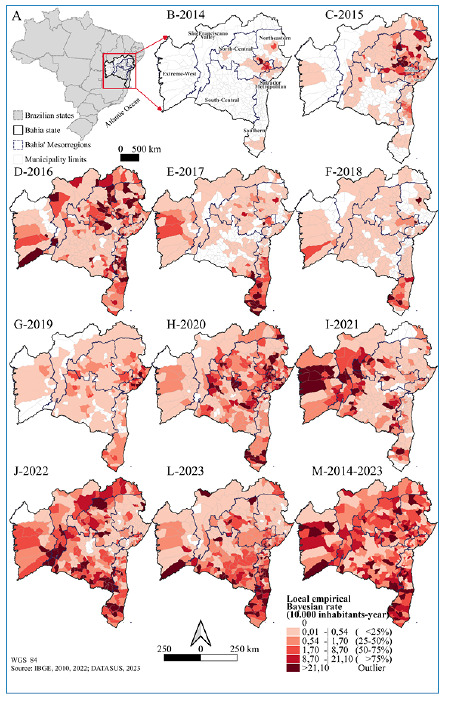
Map of Brazil and the state of Bahia (A). Evolution of the local empirical Bayesian incidence rate (10,000 inhabitants-year) of confirmed cases of chikungunya, (B-L) for each year symptom onset (2014-2023) and (M) for the entire period, according to municipalities of residence and mesoregions of Bahia.

Bahia, located in northeastern Brazil, encompasses 417 municipalities and 7 mesoregions (Northeast, Salvador Metropolitan Region, South-Central, Southern, North-Central, São Francisco Valley, and Extreme-West) (Figure 1A), covering a total area of 564,760.429 km² ^23^. Bahia has 14,141,626 inhabitants, making it the fourth most populous state in Brazil^23^. This territory exhibits significant climatic diversity, with a predominance of dry, semi-arid, and tropical climates composed of the Caatinga (53%), Cerrado (27%), and Atlantic Forest (19%) biomes^24^. It has high average temperatures, with a maximum of 30°C, and annual precipitation ranging from 1,600 mm in the coastal region to approximately 400 mm in the extreme north^25^.

### Collection of case, population, and georeferenced data

Confirmed cases, based on laboratory and clinical-epidemiological criteria, were collected from the Notifiable Diseases Information System (SINAN), of the Health Department of Bahia state between September 2014 and December 2023^26^. Data on CHIK cases were anonymized to ensure confidentiality. This study adhered to the ethical standards established in Resolutions Nos. 466 (December 12, 2012) and 580 (March 22, 2018). Population censuses, georeferenced databases for municipalities, mesoregional divisions, and the real centroids of Bahia were obtained from the Brazilian Institute of Geography and Statistics^23^^,^^27^^,^^28^.

### Data analysis

Bayesian incidence rate was calculated per 10,000 inhabitants by municipality of residence for each year of symptom onset and for the overall study period (2014-2023). Population projections were based on the 2010 and 2022 censuses, using a “Queen” contiguity neighborhood matrix. Rate categories were classified using the BoxMap 1.5 plugin (quartile and outlier) in GeoDa^29^. Monthly time series of CHIK cases by month/year were calculated using Power BI (Microsoft®).

To identify temporal, seasonal, purely spatial, and spatiotemporal clusters associated with high and low risks for CHIK, as well as clusters with spatial variations in temporal trends, Kulldorff’s spatial scan statistic was applied using SaTScan™ version 10.1.2 (Harvard Medical School, Boston, USA) was used^30^. Three datasets were used for this purpose: a) cases by municipality with month and year of symptom onset, b) population by municipality and year across the study period, and c) real municipal centroids (representing more urbanized areas). A discrete Poisson model was used, with circular scanning windows^31^.

For purely spatial analysis, the maximum population size of scanning windows was defined by the Gini index^32^^,^^33^, which captured 2% of the exposed population, optimizing the detection of the most informative non-overlapping clusters and avoiding excessively large or fragmented clusters^32^^,^^33^. This parameter was also applied in the spatiotemporal and spatial variation within the temporal trend analysis. A hierarchical model with non-overlapping secondary clusters was adopted, and the aggregation time was set to years. Relative risk (RR) was calculated for each cluster, with clusters showing p >0.05 excluded^32^^,^^34^. The p-values for the clusters were determined using Monte Carlo hypothesis testing with 999 replicates^32^. Results from SaTScan and GeoDa were imported into QGIS version 3.22.9 (QGIS Development Team, Open-Source) software to design thematic maps^35^. The Student’s t-test was used to compare the mean values between low- and high-risk clusters. Analyses were performed using R version 4.2.03 (R Core Team, Vienna, Austria), with the rstatix and car packages as described by Lacerda et al.^36^^,^^37^.

### Social and climatic variables

Information on vegetation cover for each municipality was collected using the Normalized Difference Vegetation Index (NDVI) for 2014-2023. Data was obtained from the Google Earth Engine platform and collected from a satellite at a resolution of 463 m using Python with the geemap and eemont packages^38^^-^^40^. Average NDVI values were calculated for the urban areas of each municipality as defined by the Brazilian Institute of Geography and Statistics (IBGE)^23^. 

The percentage of vegetation cover for each biome type, total coverage, and percentage of urban infrastructure were calculated using MapBiomas Collection 8 for 2014-2022^41^. Data on sewage networks, storm drainage, septic tanks, water supply, and waste collection were obtained from the 2022 IBGE census^23^. Data from the 2010 Brazilian Deprivation Index (BDI) were obtained from Cidacs/Fiocruz Bahia^42^.

Monthly precipitation and temperature data were collected from 62 meteorological stations of the National Institute of Meteorology^43^. A vector-shaped file with 62 points was created using the coordinates of each meteorological station. Columns detailing the monthly mean temperature and total precipitation data for each station over the 10-year analysis period were created using the attribute table in this shapefile. Data interpolation was performed using Quantum QGIS version 3.34.2-Prizren^35^. Monthly mean maximum and minimum temperatures, and total precipitation data were obtained from WorldClim version 2.1^44^^-^^45^, with a resolution of 2.5 min ~21 km². These averages were weighted by the area of each pixel within each municipality^46^, in QGIS. The annual and summer temperature amplitudes were calculated based on these values.

## RESULTS

### Local empirical Bayesian rates

Between 2014 and 2023, 130,344 confirmed CHIK cases were reported in 387 municipalities in Bahia (92.8%), with 30 municipalities remaining silent. More pronounced increases in disease progression were observed in the southern, north-central, and south-central mesoregions, the Salvador Metropolitan Region, and the extreme west. The southern region showed higher rates in 2017, 2018, and 2020, which declined in 2021 and recovered in 2023. The Salvador Metropolitan Region recorded the highest rates in 2019, 2020, and 2023. The extreme-west and north/south-central mesoregions showed increasing rates beginning in 2016, with another rise observed between 2021 and 2023 (Figure 1).

### Purely temporal, seasonal, and spatial analyses

**FIGURE 2:**
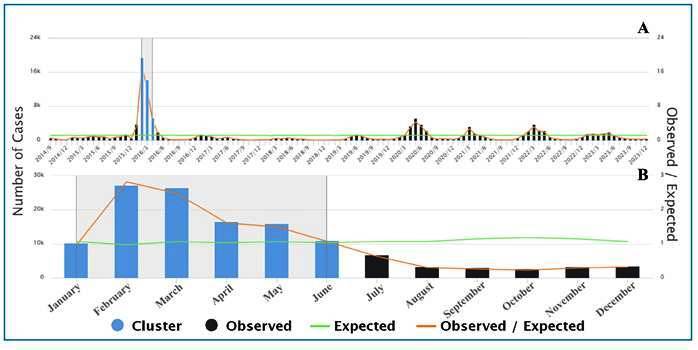
Temporal analysis by month of symptom onset (A) and seasonal analysis (B) of chikungunya cases in the state of Bahia from 2014 to 2023. Green line: expected cases under the null hypothesis of random distribution. Orange line: observed cases divided by expected, highlighting periods of higher or lower incidence.

In the purely temporal analysis, a high-risk cluster was identified between February 2015 and April 2016 (RR = 15.75), corresponding to the peak year of the disease in Bahia (Figure 2A). The months with the highest risk identified in our seasonal analysis were January and June (summer and fall; RR = 5.18), with peaks in February and March (Figure 2B).

**FIGURE 3:**
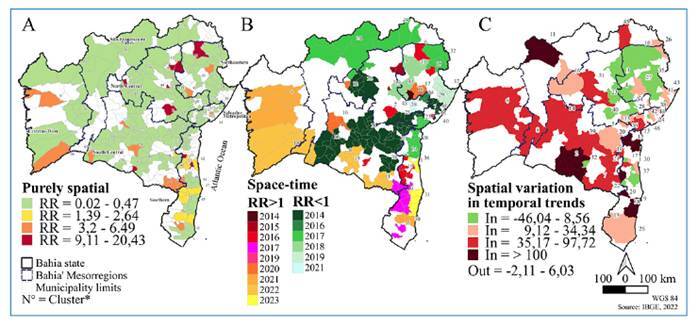
Clusters classified as high (RR> 1) and low risk (RR<1): (A) in the purely spatial analysis and; (B) temporal space by the year of cluster onset: (C) Clusters classified as high (In> 0%) and low (In<0%) trends in the analysis of the spatial variation of the temporal trends of chikungunya cases in Bahia, 2014-2023.

From the purely spatial scanning analysis, 24 high-risk clusters (RR > 1) and 38 low-risk clusters (RR < 1) were identified and distributed across 39 and 240 municipalities, respectively. The highest-risk clusters were located in the southern (Cluster 1, RR = 20.4), northeastern (Cluster 5, RR = 14.0), south-central (Cluster 6, RR = 13.9), and north-central (Cluster 2, RR = 11.8) mesoregions (Figure 3A, Supplementary Table S1, S4).

### Spatiotemporal analysis

Spatiotemporal analysis identified 44 clusters comprising 21 low-and 23 high-risk clusters. The low-risk clusters started between 2014 and 2021 and lasted for 3-5 years. They were distributed in all mesoregions, except in the extreme west. The high-risk spatiotemporal clusters between 2014 and 2015 were located in the north-central and northeastern mesoregions, whereas the clusters from 2021 to 2023 were mainly observed in the south-central, southern, and extreme-west mesoregions. The average duration of the high-risk clusters varied between 1 and 2 years; cluster 15 in the northeast region had the longest duration of 5 years (Figure 3B, Supplementary Table S2, S4). The mesoregions with the highest risk for CHIK were South (Cluster 1, 2016; RR = 179 and Cluster 14, 2016; RR = 69), North-Central (Cluster 2, 2016; RR = 119 and Cluster 13, 2015; RR = 89), and Northeast (Cluster 6, 2016; RR = 140) (Figure 3B, Supplementary Table S2, S4).

### Spatial analysis of temporal trends

Analysis of the spatial variation within CHIK temporal trends identified 46 significant clusters, of which 31 presented an internal trend of annual increase between 9.1 and 2,245.5%, while the external trend varied between -2.1 and 0.6% across the state. The largest trends were observed in the southern, south-central, and São Francisco Valley mesoregions. Fifteen clusters with decreasing internal trends were identified, located mainly in the northeastern and north-central mesoregions. The internal trend for these clusters varied between -8.5 and -46.1%, whereas the external trend varied between -0.1 and -0.6%, except for four clusters with external increasing trends of 0.5-6.0% (Figure 3C, Supplementary Table S3, S4). Overall, a decreasing annual trend of -0.6% was identified for the state.

### Student’s t-test

**FIGURE 4:**
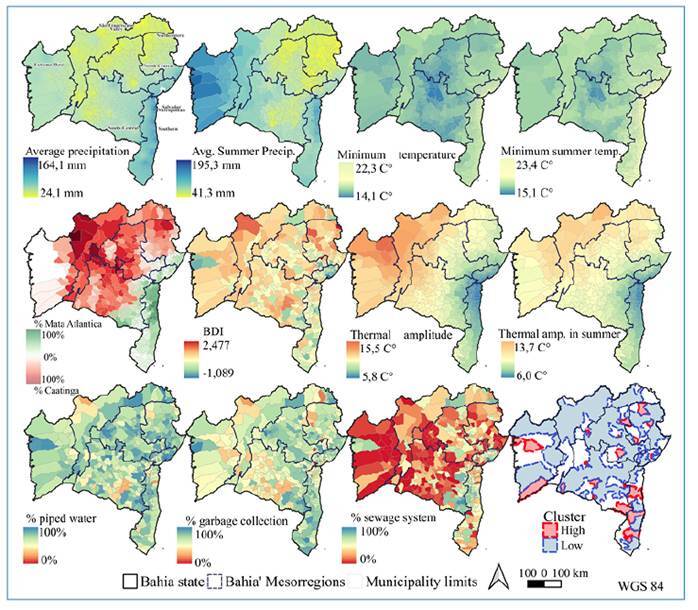
Maps of climatic, environmental, and socioeconomic variables by municipalities that were significant in the t-test, along with high-risk and low-risk areas identified in the purely spatial analysis of the state of Bahia and its mesoregions. Climatic variables: average monthly precipitation from automatic stations/INMET: interpolated data from 2014 to 2023; average summer precipitation, minimum temperature, minimum summer temperature, thermal amplitude, thermal amplitude in summer, from 2014 to 2021; Environmental variables: % of forested area of the Caatinga and Atlantic Forest biomes from 2014 to 2022; Socioeconomic variables: Brazilian Deprivation Index (BDI) for 2010, % of households with sewage system, piped water, and garbage collection from the 2022 census. Sources: IBGE^19^^,^^23^; INMET^43^; WorldClim^45^; MapBioma^41^; GEE/Vermote et al. ^39^; Cidacs/Allik et al^42^.

**SUPPLEMENTARY FIGURE S1:**
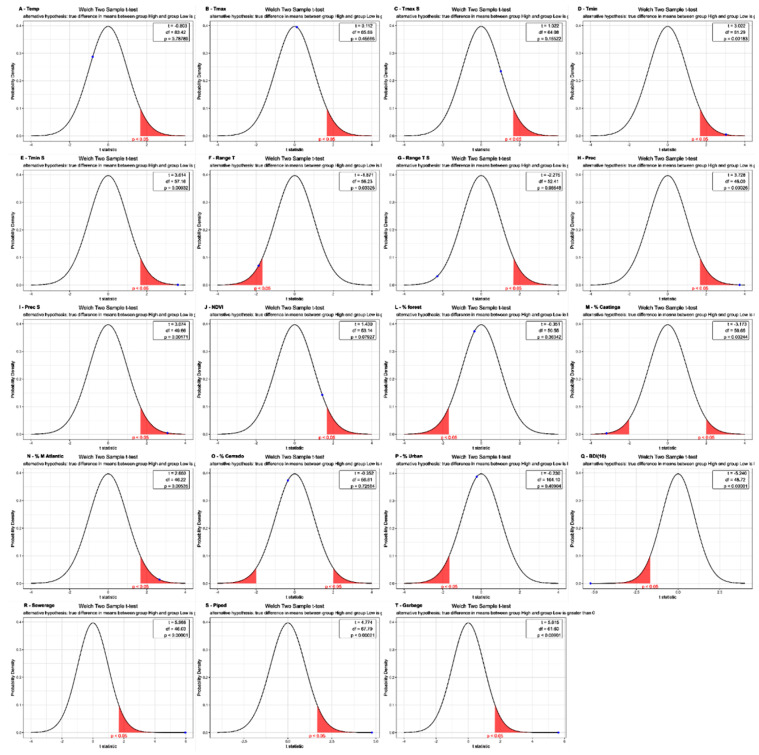
Graph of the distribution of t-test of climatic, environmental and socioeconomic variables of the municipalities of residence included in the high-risk and Low clusters chikungunya cases in the purely spatial clusters, in Bahia state, Brazil, for the period 2024-2023. Average temperature (A), Average maximum temperature (B), Average maximum temperature in summer (C), Average minimum temperature (D), Average minimum temperature in summer (E), thermal amplitude (F), thermal amplitude in summer (G), Average precipitation (H), Average precipitation in summer (I), NDVI (J), % of Forest (L), % of Caatinga' (M) , % of Mata Atlantica (N), % of Cerrado (O), % of Infra Urban (P), BDI (Q), sewage system (R), piped water (S), garbage (T).

**SUPPLEMENTARY FIGURE S2:**
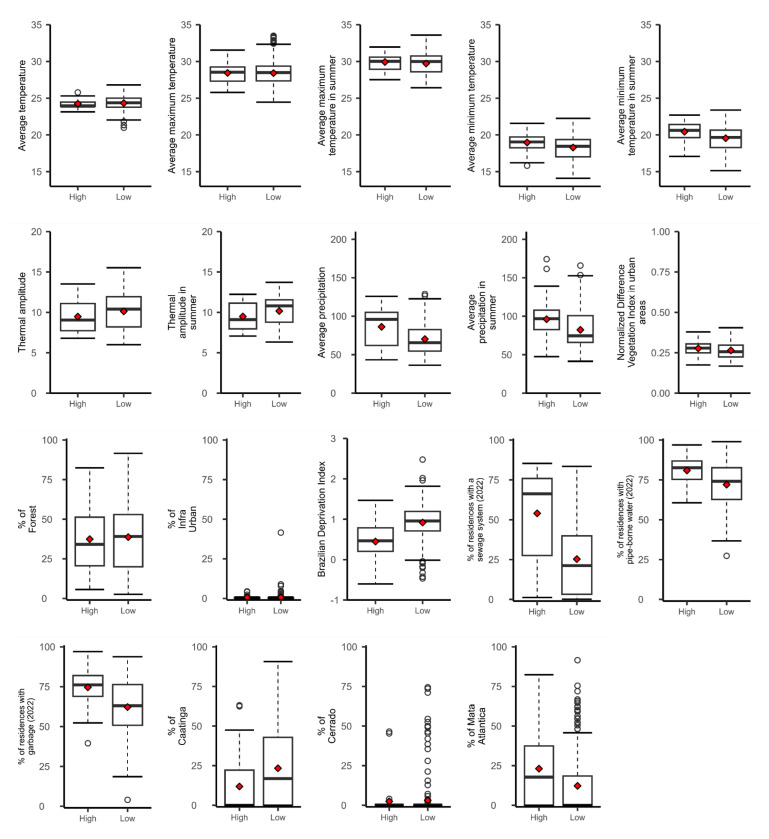
Boxplot of climatic, environmental (average of 2014-2023) and socioeconomic variables (2010, 2022) of the municipalities of residence included in the clusters of high-risk and low-risk chikungunya cases in the purely spatial clusters, for the period 2014-2023, of municipalities of Bahia state, Brazil. Point in red is the average of the group.

**SUPPLEMENTARY FIGURE S3:**
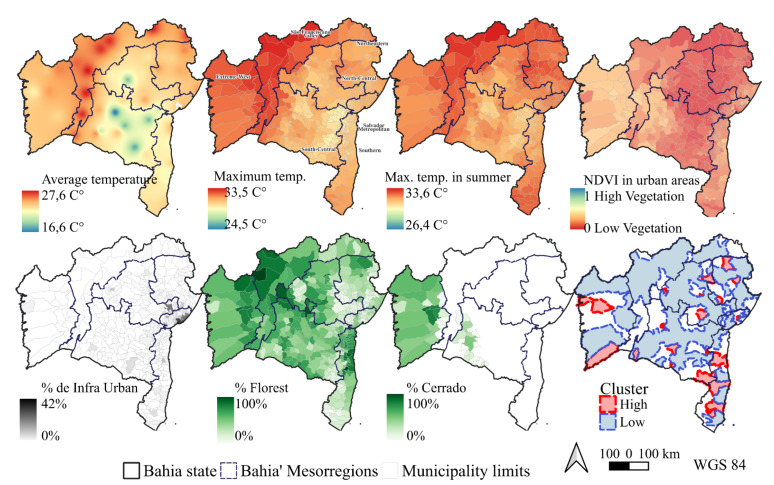
Maps of climate, environmental, and socioeconomic variables by municipalities that were not significant in the t-test, along with the high-risk and low-risk areas from the purely spatial analysis of the state of Bahia and its mesoregions.

Comparisons of the high- and low-risk municipalities included in the spatial cluster analyses are presented in Table 1. High-risk municipalities had higher annual and summer minimum temperatures, lower thermal amplitudes, and higher average monthly precipitation levels than low-risk municipalities. No significant difference was observed between high- and low-risk areas in terms of maximum and average temperatures. High-risk municipalities had higher percentages of households with piped water, garbage collection, and sewage systems, along with lower BDIs, indicating better socioeconomic conditions, along with lower percentages of vegetation cover in the Caatinga biome and higher percentages in the Atlantic Forest biome (Figure 4, Supplementary Figures S1, S2, S3).

**TABLE 1:** Comparison of climatic, environmental, and socio-economic variables between Bahia municipalities classified as high and low risk in the purely spatial scan analysis

Variable (Average)	Low risk Cluster (N=240)	High risk cluster (N=39)	t-test	p-value
Climate (2014-2023):				
Average temperature (ºC)	24.33 (1.00)	24.24 (0.56)	-0.8031	0.788
Average precipitation (mm)	70.32 (20.05)	86.24 (25.42)	3.7283	0.000*
Climate (2014-2021):				
Maximum temperature (ºC)	28.41 (1.86)	28.44 (1.32)	0.1118	0.456
Maximum temperature (ºC) in summer	29.72 (1.54)	29.93 (1.12)	1.0224	0.155
Minimum temperature (ºC)	18.28 (1.63)	18.96 (1.25)	3.0224	0.002*
Minimum temperature (ºC) in summer	19.55 (1.65)	20.44 (1.38)	3.6135	0.000*
Thermal amplitude (ºC)	10.13 (2.31)	9.47 (1.98)	-1.8712	0.033*
Thermal amplitude (ºC) in summer	10.17 (1.79)	9.49 (1.72)	-2.2749	0.014*
Average precipitation (mm) in summer	82.23 (24.84)	96.08 (26.29)	3.0738	0.002*
Environmental (2014-2023):				
NDVI in urban areas	0.26 (0.05)	0.28 (0.05)	1.4301	0.079
Environmental (2014-2022):				
% of Forest	38.7 (20.66)	37.42 (21.11)	-0.3513	0.363
% Infra Urban	0.65 (2.81)	0.59 (0.97)	-0.2304	0.409
% of Cerrado	3.1 (12.09)	2.46 (10.26)	-0.3524	0.363
% of Mata Atlântica	12.24 (19.38)	23.1 (24.25)	2.6605	0.005*
% of Caatinga	23.35 (24.08)	11.86 (20.42)	-3.1731	0.001*
Socio-economic (2010):				
Brazilian Deprivation Index (BDI)	0.91 (0.47)	0.45 (0.52)	-5.24	0.000*
Socio-economic (2022):				
Sewerage system	25.31 (22.6)	53.98 (28.59)	5.9676	0.000*
Piped water	72.01 (14.69)	80.91 (10.03)	4.7739	0.000*
Garbage collected	62.22 (16.04)	74.62 (12.19)	5.6152	0.000*

Comparisons were performed using t-tests (2014-2023). Values represent means and standard deviations. * Significant p-value < 0.05. Source: IBGE^23^, INMET^43^, WorldClin^45^, MapBioma^41^, GEE/Vermote et al.^39^, Cidacs/Allik et al.^42.^

## DISCUSSION

Significant spatial and temporal variations in CHIK incidence were identified in Bahia, reflecting the complexity of its transmission dynamics. Among the regional and annual fluctuations, greater dissemination was observed in areas of high urbanization, reinforcing the hypothesis that factors such as urbanization and mobility contribute to the spread of CHIK, such as in the municipality of Feira de Santana in the north-central mesoregion of Salvador, which was the initial focal point of CHIK. These findings suggest that the process of expansion of the disease did not occur randomly but was probably influenced by urban mobility alongside socioeconomic and environmental factors in these mesoregions^46^^-^^47^. Similar data have been observed in other studies, indicating that areas with increasing urbanization and intense human mobility tend to be more vulnerable to vector-borne disease transmissions^12^^,^^47^^-^^48^.

An association between CHIK and higher socioeconomic conditions was identified, which can be attributed to the dynamics of municipalities with higher population densities and more intense economic activities, which tend to attract a large number of individuals, consequently resulting in an increase in infections. However, some areas within these municipalities present significant economic disparities, suggesting a need for research on an intermunicipal scale that considers social heterogeneity. A comparison of socioeconomic indicators between 2010 and 2022 revealed low development levels in Bahia^23^^,^^42^. In 2010, access to basic sanitation in Bahia was limited, with 23.4% of households connected to sewage systems, 69.1% with piped water, and 49.8% with garbage collection, increasing modestly by 2022 (30.5%, 73.7%, and 64.1%, respectively^23^^,^^42^, probably contributing to the increased CHIK incidence in the state. Therefore, the association between CHIK and higher socioeconomic indicators does not negate the influence of social vulnerability on the occurrence of cases. Further research is required to confirm these associations^48^^-^^49^. Socially vulnerable populations tend to live in environments conducive to the Ae. aegypti. Inadequate water storage (due to a lack of regular water supply), accumulated garbage, and the absence of adequate urban infrastructure favor the creation of breeding grounds, increasing the risk of infection.

The association between CHIK and improved waste collection may represent a limitation of the variable itself, as it does not necessarily guarantee adequate waste disposal. Improper disposal may increase the number of water reservoirs in inappropriate locations^46^^,^^50^. High proportions of waste are disposed of in open-air dumps in Northeast Brazil, which contribute to the creation of breeding sites owing to the accumulation of water during rains, consequently leading to an increase in the vector population^50^. Furthermore, poverty is often associated with a reduced capacity for individual prevention (such as the use of screens, repellents, or water-tank sealing systems) and difficulties in accessing health services early, which can delay diagnosis and contribute to the spread of the virus.

This study demonstrated that high-risk clusters have shown higher trends in recent decades, particularly in areas with environmental degradation and substantial biodiversity loss, such as the southern, south-central, and São Francisco Valley mesoregions^51^^,^^52^. In addition to an increase in the number of CHIK cases, these regions have also shown an increase in the incidence rates of dengue and Zika. These areas are known to have a high circulation of other arboviruses, which may contribute to possible co-infections and worsening of cases^53^^,^^54^.

The association between CHIK incidence and higher percentages in the Atlantic Forest may be a limitation of the variable itself, as the Bahia Atlantic Forest is currently considered a highly fragmented area^55^. The relationship between CHIKV occurrence and biomes occurs primarily through interactions between climate, vegetation, urbanization, and local socioeconomic practices^52^^,^^53^. Densely populated regions, such as the southeast, have ideal conditions for transmission because of the combination of a hot, humid climate and high population density. The region has suffered a loss of conservation sites in recent years, resulting in a reduction in the population density of some species^55^. This scenario may contribute to continued outbreaks of arboviruses because a decrease in biodiversity may increase the population and distribution of Ae. albopictus and Ae. aegypti and the consequent risk of disease^56^^-^^57^. Conversely, an association was observed between CHIK incidence and lower percentages of Caatinga coverage, despite this biome comprising most of the state’s territory^24^. These findings underscore the complex interplay between ecological characteristics and disease dynamics, and highlight the importance of preserving biodiversity and ecosystem integrity in mitigating arbovirus risk.

The removal of large areas of native vegetation leads to declines in natural ecosystems, which can manifest as soil erosion and changes in the course of watersheds^58^, which can produce fluctuations in wind, water, and temperature, affecting the spatial dynamics of the local vector Ae. albopictus^58^. Furthermore, anthropogenic actions in conservation areas can create environments with greater interactions between human hosts and vectors, in addition to increasing the number of artificial breeding sites, thereby contributing to an increase in Ae. aegypti. According to Abdullah^59^, vectors prefer to lay eggs in artificial containers rather than in natural vegetation. This finding is consistent with that of Vieira^58^, who reported an increase in these species under conditions of greater anthropogenic influence. Further, biodiversity preservation is associated with lower risks of arbovirus transmission because of the preservation of native species, which would serve as a food source for vectors, reducing human-animal contact and the risk of disease transmission. In this context, the preservation of native vegetation can contribute to the control of arbovirus disease outbreaks in the state^56^^,^^60^.

The analysis revealed an increase in cases in February and March, differing from the trend in Brazil, where the peak occurred between April and May^46^. This finding serves as a warning to surveillance services regarding possible outbreaks between late summer and early fall, which is the period of the greatest epidemiological alert. This period was favorable for the intensification of socio-educational campaigns and vector control measures in the state. 

Climatic variables can explain both the spatial distribution of arboviral disease incidence and its variation over time. Areas beyond the Agreste (semi-arid transitional zone) and Sertão (semi-arid hinterlands) of Bahia are generally warmer, and thus present higher risks of arboviruses because elevated temperatures increase their transmission potential^61^. The climate in these regions is generally dry for most of the year, and the rainy season is more intense in summer^25^. However, in certain municipalities within the central-west region, temperatures remained constant throughout the year, and precipitation levels increased only during winter^21^. Research indicates that precipitation, especially in the months following heavy rainfall, combined with temperature peaks, creates favorable conditions for mosquito survival and reproduction, increasing vector density and contributing to arbovirus outbreaks^53^^,^^59^^,^^62^^,^^63^. Furthermore, prolonged periods of drought in semiarid regions contribute to the storage of water in cisterns, thereby increasing the number of potential breeding sites^25^. Therefore, the intensification of socioeducational actions related to adequate water storage can contribute to vector control in these areas. The observed association between CHIK and elevated minimum temperatures is consistent with previous evidence that arbovirus transmission in tropical regions is particularly sensitive to variations in minimum temperatures^59^^,^^61^^-^^65^, probably because both the mean and maximum temperatures generally remained constant in these regions, with greater variation in minimum temperatures^59^. Warmer climates may increase vector densities by accelerating reproductive cycles, shortening egg latency, and reducing the extrinsic incubation period of the virus^62^^,^^64^, while also influencing human behavior, such as greater skin exposure^65^. According to the Intergovernmental Panel on Climate Change (IPCC), even a 1°C increase in global temperature may significantly amplify the risk of arbovirus transmission^66^. Although the temperature oscillations identified in this study were small, they may still have epidemiological relevance when combined with local biomes, urbanization, and social vulnerability. Therefore, understanding these climatic aspects is crucial for a better understanding of the epidemiological characteristics of CHIK in Bahia. 

Although the analysis indicates a decreasing annual trend in the state, the possibility of new outbreaks cannot be ruled out. In addition to CHIKV, which has the potential for genetic mutation^67^^-^^68^, changes in terrestrial climate patterns have contributed to the global increase in several diseases, including those caused by arboviruses^61^^,^^69^. In addition, this climate scenario indicates an increase in the vector density and geographic expansion of arboviruses, thus increasing the risk of morbidity and mortality associated with these diseases^2^^,^^16^. Consequently, these regions will continue to be at risk of new CHIK outbreaks that require monitoring. 

This study has some limitations, including the use of secondary CHIK data, the limited number of climate stations in Bahia, and the potential for underreporting due to diagnostic challenges in the context of co-circulation of chikungunya, dengue, and Zika. Associations are ecological and do not imply direct causation because socioeconomic factors are likely to interact with local climatic conditions and influence vector proliferation and transmission. Although weather stations in Bahia are unevenly distributed, this was mitigated using WorldClim, which combines station data, satellite observations, and spatial modeling to estimate climate variables in areas without direct measurements. However, implementation and investment in additional meteorological stations are essential to further improve data accuracy and support more detailed local analyses.

Despite these limitations, our findings are useful for identifying high-risk areas, guiding outbreak prevention, and advancing existing knowledge. They also provide a foundation for more robust future studies using higher spatial and temporal resolution data, such as high-resolution satellite imagery and spatiotemporal Bayesian modeling, to deepen the understanding of the interactions between climatic, socioeconomic, and epidemiological factors and to identify critical micro-areas. 

Furthermore, this study highlighted that increases in minimum temperature and precipitation levels may influence the distribution of the disease and that socioeconomic factors should be further investigated at the inter-municipal, intra-urban, or census sector scale to properly consider the high levels of social disparity across municipalities in Bahia. Prevention and epidemiological control measures should continue throughout the year and intensify during the summer and fall.

In this context, the synergy between technological innovation and traditional knowledge can lead to a more effective control of arboviral diseases^70^. The combination of traditional methods, such as the elimination of breeding sites and the use of insecticides, with modern technologies, including genetic engineering of mosquitoes and mosquito infection with Wolbachia, represents an integrated strategy that enhances the effectiveness of combating these infections^70^^-^^71^. Furthermore, engaging local communities is essential for increasing the awareness and uptake of prevention strategies^70^.

This study identified spatial and temporal clusters of high-risk CHIK in Bahia and associated them with several environmental and social factors. An overall declining trend in the incidence of -0.6% per year was observed. However, the persistence of high-risk clusters in several states justifies the need for continuous disease surveillance to prevent outbreaks.

Social education, prevention, and control measures implemented by epidemiological services in Bahia must continue throughout the year and intensify during the summer and autumn. Alongside these efforts, political actions focusing on climate change mitigation, adaptation, and multivariate community education initiatives paired with improvements in urban infrastructure may contribute to reducing risk.

## DATA AVAILABILITY STATEMENT

 Data-available: Secondary data on chikungunya were obtained from Brazil’s Notification Disease Information System (SINAN) is available by the Health Department of the state of Bahia (SESAB) repository(https://www.saude.ba.gov.br/suvisa/vigilancia-epidemiologica/agravos-morbidade-epidemiologia/).

Additional datasets used include:

 IBGE: Municipal boundaries, mesoregional divisions https://www.ibge.gov.br/geociencias/downloads-geociencias.html); real centroids (https://www.ibge.gov.br/geociencias/cartas-e-mapas/redes-geograficas/15789-areas-urbanizadas.html?=&t=downloads); population, and socioeconomic factors (https://www.ibge.gov.br/estatisticas/sociais/populacao/).

 Google Earth Engine (GEE): NDVI (https://developers.google.com/earth-engine/datasets/catalog/MODIS_MOD09GA_006_NDVI).

 MapBiomas: Environmental variables (https://brasil.mapbiomas.org/estatisticas/).

 Cidacs/Fiocruz Bahia: Brazilian Deprivation Index (BDI) (https://cidacs.bahia.fiocruz.br/ibp/).

 Climatic data: INMET (https://portal.inmet.gov.br/) and WorldClim (https://www.worldclim.org/data/monthlywth.html).

 Data-in-article: Processed data analyzed during the current study are included in this published article and its supplementary information files (Supplementary Table S1 and S4).

## Figures and Tables

Climate Variables: Average temperature from automatic weather stations/INMET: interpolated data from the period of 2014 to 2023; maximum temperature, maximum temperature in summer, from the period of 2014 to 2021.

Environmental Variables: Average NDVI from the period of 2014 to 2023, % of forested area in the municipality, % of forested area of the Cerrado type, % of area with urban infrastructure of the total area of the municipality from the period of 2014 to 2022.

Source: IBGE, 2022, 2024; INMET, 2022, WorldClin, 2023, MapBioma, 2023, GEE/Vermote et al., 2024.

## Appendix

**SUPPLEMENTARY TABLE S1:** Purely Spatial Analysis’ clusters of chikungunya cases in Bahia, 2014-2023.

Cluster	Nº M	Observed	Expected	RR	Population	P value
1	1	28596	1768.86	20.43	191463.25	0.001
2	1	6646	592.76	11.76	64161.61	0.001
3	1	7687	1324.78	6.10	143395.61	0.001
4	2	4253	480.93	9.11	52056.03	0.001
5	1	2038	147.88	13.98	16006.24	0.001
6	1	1288	93.43	13.91	10113.43	0.001
7	5	2755	614.46	4.56	66509.67	0.001
8	1	1972	307.92	6.49	33329.34	0.001
9	1	4300	1421.15	3.09	153827.47	0.001
10	10	249	2592.52	0.09	280617.39	0.001
11	15	133	2261.98	0.06	244839.88	0.001
12	18	266	2465.20	0.11	266835.91	0.001
13	16	337	2558.28	0.13	276911.76	0.001
14	1	1159	124.57	9.38	13483.10	0.001
15	1	2100	483.10	4.40	52291.03	0.001
16	6	127	1874.07	0.07	202851.64	0.001
17	1	2496	695.07	3.64	75235.31	0.001
18	15	343	2376.93	0.14	257281.91	0.001
19	4	104	1690.72	0.06	183005.33	0.001
20	7	140	1745.29	0.08	188912.70	0.001
21	10	502	2564.43	0.19	277577.07	0.001
22	3	3163	1165.96	2.76	126204.52	0.001
23	3	569	2508.61	0.22	271535.08	0.001
24	12	509	2374.02	0.21	256967.01	0.001
25	1	721	76.21	9.51	8249.28	0.001
26	12	395	1973.36	0.20	213598.97	0.001
27	5	72	1115.81	0.06	120776.17	0.001
28	2	50	1035.50	0.05	112084.19	0.001
29	1	1907	636.96	3.02	68945.66	0.001
30	5	94	1149.95	0.08	124472.23	0.001
31	7	791	2423.45	0.32	262317.82	0.001
32	11	894	2575.82	0.34	278810.60	0.001
33	1	668	94.79	7.08	10259.94	0.001
34	12	553	1905.27	0.29	206229.16	0.001
35	1	507	1780.94	0.28	192770.90	0.001
36	9	240	1172.73	0.20	126938.33	0.001
37	1	560	100.45	5.59	10873.26	0.001
38	11	1130	2479.55	0.45	268389.39	0.001
39	3	31	511.88	0.06	55406.11	0.001
40	6	330	1124.23	0.29	121688.00	0.001
41	1	304	1066.90	0.28	115482.56	0.001
42	1	41	521.85	0.08	56485.43	0.001
43	3	165	788.75	0.21	85374.94	0.001
44	3	41	499.17	0.08	54030.83	0.001
45	3	938	1977.07	0.47	214000.90	0.001
46	4	80	579.83	0.14	62761.98	0.001
47	3	221	773.50	0.28	83724.56	0.001
48	1	557	174.49	3.20	18886.75	0.001
49	7	346	956.30	0.36	103511.41	0.001
50	4	79	475.79	0.17	51500.30	0.001
51	1	14	301.10	0.05	32591.12	0.001
52	2	255	748.50	0.34	81018.88	0.001
53	1	378	102.18	3.71	11060.28	0.001
54	2	6	237.66	0.03	25724.37	0.001
55	8	1565	907.53	1.73	98232.44	0.001
56	2	1480	864.48	1.72	93572.17	0.001
57	1	501	189.99	2.64	20565.04	0.001
58	2	4	165.54	0.02	17917.95	0.001
59	1	306	104.89	2.92	11353.80	0.001
60	3	131	388.38	0.34	42039.22	0.001
61	1	10	143.76	0.07	15561.11	0.001
62	1	1852	1664.14	1.11	180128.35	0.016

Cluster = Cluster identification number; see Fig. 3A. Nº M = Number of Municipalities.

## Appendix

**SUPPLEMENTARY TABLE S2:** Space-time analysis’ clusters of chikungunya cases in Bahia, 2014-2023.

C*	Start - End Date		Duration (Year)	Nº M	Observed	Expected	RR	Pop	p-value
1	2016/1/1	2016/12/31	1	1	26039	181.22	179.31	191941.33	0.001
2	2016/1/1	2016/12/31	1	1	6633	58.67	119.06	64069.99	0.001
3	2015/1/1	2016/12/31	2	2	4236	96.84	45.18	52084.88	0.001
4	2021/1/1	2021/12/31	1	8	5170	249.14	21.57	264007.15	0.001
5	2017/1/1	2018/12/31	2	2	4564	279.26	16.90	151834.68	0.001
6	2016/1/1	2016/12/31	1	1	2038	14.74	140.42	15996.31	0.001
7	2019/1/1	2019/12/31	1	1	2393	69.34	35.14	75522.17	0.001
8	2022/1/1	2023/12/31	2	9	3852	317.76	12.46	172627.73	0.001
9	2017/1/1	2017/12/31	1	8	2716	198.41	13.96	215575.32	0.001
10	2014/1/1	2015/12/31	2	1	1811	61.42	29.88	33323.64	0.001
11	2022/1/1	2022/12/31	1	6	2048	100.50	20.69	108943.89	0.001
12	2020/1/1	2020/12/31	1	2	1364	26.27	52.46	28216.10	0.001
13	2015/1/1	2015/12/31	1	1	690	7.78	89.12	8264.19	0.001
14	2016/1/1	2016/12/31	1	1	656	9.49	69.50	10258.02	0.001
15	2016/1/1	2020/12/31	5	1	1802	239.69	7.61	52118.20	0.001
16	2020/1/1	2021/12/31	2	4	1506	160.45	9.48	88348.97	0.001
17	2020/1/1	2020/12/31	1	8	1472	204.80	7.26	219923.43	0.001
18	2022/1/1	2022/12/31	1	11	1463	206.80	7.14	222523.67	0.001
19	2016/1/1	2016/12/31	1	8	867	72.13	12.09	76168.67	0.001
20	2014/1/1	2018/12/31	5	10	5	1298.65	0.00	280925.82	0.001
21	2014/1/1	2018/12/31	5	17	13	1259.47	0.01	274996.98	0.001
22	2014/1/1	2018/12/31	5	11	17	1276.22	0.01	278513.68	0.001
23	2017/1/1	2021/12/31	5	10	36	1296.32	0.03	280710.83	0.001
24	2017/1/1	2021/12/31	5	8	31	1239.79	0.02	267662.24	0.001
25	2014/1/1	2018/12/31	5	18	31	1236.17	0.02	266917.24	0.001
26	2014/1/1	2018/12/31	5	11	48	1273.58	0.04	277568.58	0.001
27	2017/1/1	2021/12/31	5	3	46	1257.52	0.04	270424.65	0.001
28	2017/1/1	2021/12/31	5	13	46	1245.01	0.04	269434.24	0.001
29	2014/1/1	2018/12/31	5	8	66	1258.05	0.05	272906.46	0.001
30	2021/1/1	2022/12/31	2	2	1030	174.46	5.94	93468.03	0.001
31	2023/1/1	2023/12/31	1	3	986	203.88	4.87	205206.23	0.001
32	2017/1/1	2021/12/31	5	13	101	1110.37	0.09	240855.63	0.001
33	2018/1/1	2022/12/31	5	5	25	872.87	0.03	187749.45	0.001
34	2018/1/1	2022/12/31	5	14	62	988.19	0.06	212826.16	0.001
35	2016/1/1	2016/12/31	1	1	301	9.34	32.31	10022.18	0.001
36	2023/1/1	2023/12/31	1	1	871	164.62	5.32	180277.08	0.001
37	2020/1/1	2023/12/31	4	11	74	930.02	0.08	254353.13	0.001
38	2014/1/1	2018/12/31	5	7	55	874.28	0.06	188422.73	0.001
39	2014/1/1	2018/12/31	5	7	22	755.87	0.03	161817.16	0.001
40	2021/1/1	2023/12/31	3	1	17	563.02	0.03	191709.22	0.001
41	2014/1/1	2018/12/31	5	2	4	509.88	0.01	111886.45	0.001
42	2014/1/1	2018/12/31	5	1	57	537.33	0.11	115575.42	0.001
43	2021/1/1	2023/12/31	3	5	9	284.58	0.03	101019.33	0.001
44	2016/1/1	2019/12/31	4	4	9	265.32	0.03	71866.77	0.001

*C = Clusters’ identification number, see Fig. 3A. Nº M = Number of municipalities.

## Appendix

**SUPPLEMENTARY TABLE S3:** Spatial variation in temporal trends analysis’ clusters of chikungunya cases in Bahia, 2014-2023.

*Cluster	Nº M	Observed	Expected	RR	Population	Trend (%)		p-value
						In	Out	
1	1	28596	1773.69	20.37	191941.33	-22.35	6.03	0.001
2	17	4158	2490.43	1.69	269503.43	131.01	-2.11	0.001
3	9	5136	2292.21	2.29	248053.02	-36.72	0.79	0.001
4	16	3500	2185.45	1.62	236500.62	-46.04	0.49	0.001
5	1	6646	592.06	11.77	64069.99	-30.76	0.98	0.001
6	9	5379	2529.10	2.18	273688.93	42.54	-1.91	0.001
7	8	2842	1693.45	1.69	183257.94	74.62	-1.49	0.001
8	8	1663	2303.45	0.72	249269.67	72.04	-1.11	0.001
9	3	938	1966.69	0.47	212827.11	97.72	-0.90	0.001
10	5	2132	1730.37	1.24	187253.47	-30.20	-0.13	0.001
11	2	384	596.48	0.64	64548.25	2245.56	-0.77	0.001
12	11	596	1868.75	0.32	202228.81	151.23	-0.83	0.001
13	11	1206	2457.36	0.49	265924.78	56.94	-0.94	0.001
14	15	1944	2564.94	0.75	277567.51	35.17	-1.02	0.001
15	8	10039	2521.44	4.23	272859.25	12.00	-1.58	0.001
16	13	2529	2531.57	1.00	273956.20	28.91	-1.08	0.001
17	3	690	400.98	1.72	43392.23	71.38	-0.82	0.001
18	1	318	88.33	3.61	9558.95	224.48	-0.74	0.001
19	13	1049	2283.33	0.46	247092.81	41.63	-0.86	0.001
20	10	885	2600.85	0.34	281453.47	34.34	-0.80	0.001
21	1	668	94.79	7.08	10258.02	-29.02	-0.47	0.001
22	2	210	107.83	1.95	11669.14	104.31	-0.69	0.001
23	3	333	453.03	0.73	49025.25	58.48	-0.71	0.001
24	6	3578	2323.97	1.55	251490.78	11.67	-0.93	0.001
25	4	750	1076.44	0.69	116488.24	30.30	-0.75	0.001
26	3	953	1306.64	0.73	141399.10	22.32	-0.76	0.001
27	6	1634	2273.28	0.72	246005.07	15.86	-0.80	0.001
28	1	391	342.98	1.14	37115.71	-28.20	-0.53	0.001
29	1	369	90.58	4.08	9801.86	-27.91	-0.54	0.001
30	1	1852	1665.90	1.11	180277.08	12.46	-0.79	0.001
31	3	273	607.69	0.45	65761.74	42.67	-0.68	0.001
32	1	305	92.61	3.30	10022.18	-29.32	-0.55	0.001
33	6	538	1155.84	0.46	125080.41	-20.38	-0.53	0.001
34	1	238	312.31	0.76	33797.07	-27.34	-0.57	0.001
35	10	2615	2529.76	1.03	273760.12	-8.56	-0.45	0.001
36	6	641	1586.09	0.40	171639.94	16.75	-0.69	0.001
37	4	213	455.47	0.47	49289.59	-25.65	-0.58	0.001
38	3	247	626.91	0.39	67841.71	-22.36	-0.58	0.001
39	11	107	1683.61	0.06	182193.17	38.81	-0.64	0.001
40	6	119	971.14	0.12	105092.91	25.24	-0.63	0.001
41	1	37	78.74	0.47	8520.61	-30.28	-0.61	0.001
42	2	91	132.21	0.69	14307.39	19.69	-0.63	0.002
43	4	174	851.52	0.20	92148.21	12.72	-0.64	0.009
44	1	304	1068.01	0.28	115575.42	9.12	-0.64	0.011
45	1	23	310.43	0.07	33593.70	46.23	-0.62	0.016
46	1	22	185.39	0.12	20061.75	44.72	-0.62	0.024

*Cluster = Clusters’ identification number, see Fig. 3C. Nº M = Number of municipalities.

## References

[1] 1. Bartholomeeusen K, Daniel M, LaBeaud DA, Gasque P, Peeling RW, KE Stephenson KE et al. Febre Chikungunya. Nat Rev Dis Primers. 2023;9(17). Available from: https://doi.org/10.1038/s41572-023-00429-2 10.1038/s41572-023-00429-2 PMC11126297 37024497

[2] 2. Sant’Anna MW, Ferreira ML, da Silva LF, Côrtes PL. Climate Change and Arbovirus: A Review and Bibliometric Analysis. Climate. 2025,13(35)1-21. Available from: https://doi.org/10.3390/cli13020035

[3] 3. Vairo F, Haider N, Kock R, Ntoumi F, Ippolito G, Zumla A. Chikungunya: epidemiology, pathogenesis, clinical features, management, and prevention. Infect Dis Clin North Am. 2019;33(4):1003-25. Available from: https://doi.org/10.1016/j.idc.2019.08.006. 10.1016/j.idc.2019.08.006 31668189

[4] 4. SESAB - Secretaria de Saúde do Estado da Bahia. Boletim Epidemiológico Nº 02. Atualização da distribuição de Aedes aegypti e Aedes albopictus na Bahia. [Internet]. 2016. Accessed Mar 25, 2024 Available from: Available from: https://www.saude.ba.gov.br/wp-content/uploads/2017/08/Boletim_Entomologico_Aedes_n02_10junho2016.pdf

[5] 5. Lima-Camara TN. Arboviroses emergentes e novos desafios para a saúde pública no Brasil. Rev Saúde Pública. 2016;50:36. Available from: https://doi.org/10.1590/S1518-8787.2016050006791.

[6] 6. Carabali M, Harper S, Lima-Neto AS, Sousa GS, Caprara A, Restrepo BN, Kaufman JS. Spatiotemporal distribution and socioeconomic disparities of dengue, chikungunya and Zika in two Latin American cities from 2007 to 2017. Trop Med Int Health. 2021;26(3):301-15. Available from: https://doi.org/10.1111/tmi.13530. 10.1111/tmi.13530 33219561

[7] 7. Prophiro JS. Arboviroses e mudanças climáticas. Rev Gestão Sustentabilidade Ambient. 2022;11(1):1-2. Available from: https://portaldeperiodicos.animaeducacao.com.br/

[8] 8. Sharif N, Sarkar MK, Ferdous RN, Ahmed SN, Billah BM, Talukder AA, et al. Molecular Epidemiology, Evolution and Reemergence of Chikungunya Virus in South Asia. Front Microbiol. 2021;12:689979. Available from: https://doi.org/10.3389/fmicb.2021.689979 10.3389/fmicb.2021.689979 PMC8215147 34163459

[9] 9. Cavalcanti TYVL, Pereira MR, de Paula SO, Franca RFO. A Review on Chikungunya Virus Epidemiology, Pathogenesis and Current Vaccine Development. Viruses. 2022;14(5):969. Available from: https://doi.org/10.3390/v14050969 10.3390/v14050969 PMC9147731 35632709

[10] 10. Battisti V, Urban E, Langer T. Antivirals against the Chikungunya virus. Viruses. 2021;13(7):1307. Available from: https://doi.org/10.3390/v13071307 10.3390/v13071307 PMC8310245 34372513

[11] 11. Freitas ARR, Donalisio MR, Alarcón-Elbal PM. Excess mortality and causes associated with chikungunya, Puerto Rico, 2014-2015. Emerg Infect Dis. 2018;24(12):2352-55. Available from: https://doi.org/10.3201/eid2412.170639 10.3201/eid2412.170639 PMC6256393 30277456

[12] 12. Lima STS, de Souza WM, Cavalcante JW, da Silva Candido D, Fumagalli MJ, Carrera JP, et al. Fatal outcome of chikungunya virus infection in Brazil. Clin Infect Dis. 2021;73(7):e2436-43. Available from: https://doi.org/10.1093/cid/ciaa1038 10.1093/cid/ciaa1038 PMC8492446 32766829

[13] 13. Cerqueira-Silva T, Pescarini JM, Cardim LL, Barral-Netto M, Barreto ML, Teixeira MG, et al. Risk of death following chikungunya virus disease in the 100 Million Brazilian Cohort, 2015-2018: a matched cohort study and self-controlled case series. Lancet Infect Dis. 2024;24(5):504-13. Available from: https://doi.org/10.1016/S1473-3099(23)00739-9 10.1016/S1473-3099(23)00739-9 38342106

[14] 14. Freitas ARR, Lima Neto AS, Rodrigues R, Oliveira EA, Andrade Jr JS. Excess mortality associated with chikungunya epidemic in Southeast Brazil, 2023. Front Trop Dis. 2024;5:1466207. Available from: https://doi.org/10.3389/fitd.2024.1466207

[15] 15. Jaquet M, Bengue M, Lambert K, Carnac G. Human muscle cells sensitivity to chikungunya virus infection relies on their glycolysis activity and differentiation stage. Biochimie. 2024;218:85-95. Available from: https://doi.org/10.1016/j.biochi.2023.09.005 10.1016/j.biochi.2023.09.005 37716499

[16] 16. IPCC. Intergovernmental Panel on Climate Change Printed. Policymakers [Internet]. 2022. Accessed Apr 16, 2024. Available from: Available from: https://www.ipcc.ch/

[17] 17. SESAB - Secretaria de Saúde do Estado da Bahia. Notícias: Casos de chikungunya crescem 434% na Bahia entre 2019 e 2020. SESAB [Internet]. 2020. Accessed Aug 17, 2022. Available from: Available from: http://www.saude.ba.gov.br/2020/07/06/casos-de-chikungunya-crescem-434-na-bahia-entre-2019-e-2020/

[18] 18. Silva FCM, Bezerra HDS, Araújo AOC, Carvalho LES, Silva JA. Estudo temporal das arboviroses: Uma análise espacial. Pesqui Soc Desenvolv. 2021;10(7):e10910716220. Available from: https://doi.org/10.33448/rsd-v10i7.16220

[19] 19. Santana LS, Braga JU. Spatial diffusion of Zika fever epidemics in the Municipality of Salvador-Bahia, Brazil, in 2015-2016: does Zika fever have the same spread pattern as Dengue and Chikungunya fever epidemics?. Rev Soc Bras Med Trop. 202053:e20190563. Available from: https://doi.org/10.1590/0037-8682-0563-2019 10.1590/0037-8682-0563-2019 PMC7156255 32267460

[20] 20. Sharif N, Sarkar MK, Ferdous RN, Ahmed SN, Billah BM, Talukder AA, et al. Molecular Epidemiology, Evolution and Reemergence of Chikungunya Virus in South Asia. Front Microbiol. 2021;12:689979. Available from: https://doi.org/10.3389/fmicb.2021.689979 10.3389/fmicb.2021.689979 PMC8215147 34163459

[21] 21. Souza JHM, Barros TB, Almeida PP, Vieira SCA, Melo FF, Silva RAA, Tomazi L. Dynamics of transmission of urban arbovirus dengue, Zika and Chikungunya in southwestern region of Bahia, Brazil. An Acad Bras Cienc. 2021;93(3):e20200670. Available from: https://doi.org/10.1590/0001-3765202120200670 10.1590/0001-3765202120200670 33681889

[22] 22. Argibay HD, Cardoso CW, Souza WM, Souza RL, Pellizzaro M, Cunha GM, et al. High-resolution spatiotemporal analysis of chikungunya epidemics between 2019 and 2020 in Salvador, Brazil: a municipality-level transmission dynamics study. Lancet Reg Health Am., 2025(43):101003. Available from: 10.1016/j.lana.2025.101003 PMC11804771 39925861

[23] 23. IBGE - Instituto Brasileiro de Geografia e Estatística. Censo demográfico 2010 e 2022. Características da população e dos domicílios: resultados do universo. Rio de Janeiro: IBGE; 2024. Available from: https://www.ibge.gov.br/estatisticas/sociais/populacao [dataset]

[24] 24. Dutra AC. Mapeamento e monitoramento da cobertura vegetal do estado da Bahia utilizando dados multitemporais de sensores ópticos orbitais. São José dos Campos: Instituto Nacional de Pesquisas Espaciais; 2019. Available from: http://urlib.net/sid.inpe.br/mtc-m21c/2019/03.25.13.40

[25] 25. Medauar CC, Silva SA, Carvalho LCC, Galvão IM & Macedo PV. Spatial-temporal variability of rainfall and mean air temperature for the state of Bahia, Brazil. An Acad Bras Cienc. 2020;92:e20181283. Available from: https://doi.org/10.1590/0001-3765202020181283 10.1590/0001-3765202020181283 32321014

[26] 26. SESAB - Secretaria de Saúde do Estado da Bahia. Agravos. Morbidade e Epidemiologia. Indicadores/TABNET [Internet]. 2024. Accessed Jan, 2024. Available from: Available from: https://www.saude.ba.gov.br/suvisa/vigilancia-epidemiologica/agravos-morbidade-epidemiologia/ [dataset]

[27] 27. IBGE - Instituto Brasileiro de Geografia e Estatística. Malha de setores censitários, municipalities, mesoregional divisions: Brasil [Internet]. 2022. Accessed Jan, 2023. Available from: Available from: https://www.ibge.gov.br/geociencias/downloads-geociencias.html [dataset]

[28] 28. IBGE - Instituto Brasileiro de Geografia e Estatística. Áreas urbanizadas do Brasil: 2019. IBGE; 2019. Available fromhttps://www.ibge.gov.br/geociencias/cartas-e-mapas/redes-geograficas/15789-areas-urbanizadas.html?=&t=downloads[dataset]

[29] 29. Anselin L, Rey SJ. Modern Spatial Econometrics in Practice, a Guide to GeoDa, GeoDaSpace and PySAL. Chicago, IL: GeoDa Press; 2014. Available from: https://geodacenter.github.io/GeoDaSpace/ [software]

[30] 30. Kulldorff M. Information Management Services Inc. SaTScan-Software for the spatial, temporal, and space-time scan statistics. Version 10.0.2 [Internet]. 2022. Accessed, 2025. Available from: Available from: http://www.satscan.org/ [software]

[31] 31. Yu Y, Wu B, Wu C, Wang Q, Hu D, Chen W. Spatial-temporal analysis of tuberculosis in Chongqing, China 2011-2018. BMC Infect Dis. 2020;20:531. Available from: https://doi.org/10.1186/s12879-020-05249-3 10.1186/s12879-020-05249-3 PMC7374877 32698763

[32] 32. Kulldorff M. SaTScanTM Manual do Usuário, versão 10.0 Edition, versão Traduzida para o Português por Lacerda AB, Bermudi PMM, Pellini ACG, 2022 (2021). Available from: http://www.satscan.org/

[33] 33. Han J, Zhu L, Kulldorff M, Hostovich S, Stinchcomb DG, Tatalovich Z, et al. Using Gini coefficient to determine optimal cluster reporting sizes for spatial scan statistics. Int J Health Geogr. 2016;15(1):1-11. Available from: https://doi.org/10.1186/s12942-016-0056-6 10.1186/s12942-016-0056-6 PMC4971627 27488416

[34] 34. Kulldorff M. Spatial scan statistics: models, calculations, and applications. 1999.

[35] 35. QGIS. Quantum Geographic Information System Versão 3.34.2-Prizren. Open Source Geospatial Foundation Project [Internet]. 2023. Accessed 16 Apr, 2023. Available from: Available from: http://qgis.osgeo.org/ [software]

[36] 36. R Core Team. R version 4.2.0: A language and environment for statistical computing [Internet]. 2022. Accessed, 2022. Available from: Available from: https://www.r-project.org/ [software]

[37] 37. Lacerda AB, Lorenz C, Azevedo TS, Cândido DM, Wen FH, Eloy LJ, Chiaravalloti-Neto F. Detection of areas vulnerable to scorpionism and its association with environmental factors in São Paulo, Brazil. Acta Trop. 2022;230:106390. Available from: https://doi.org/10.1016/j.actatropica.2022.106390 10.1016/j.actatropica.2022.106390 35245492

[38] 38. Wu Q. geemap: A Python package for interactive mapping with Google Earth Engine. J Open Source Softw. 2020;5(51):2305. Available from: https://doi.org/10.21105/joss.02305

[39] 39. Vermote E, Wolfe R. MOD09GA MODIS/Terra Surface Reflectance Daily L2G Global 1km and 500m SIN Grid V006 [Internet]. NASA EOSDIS Land Processes DAAC; 2015. https://doi.org/10.5067/MODIS/MOD09GA.00 GEE. Earth Engine Data Catalog. Accessed Apr 16, 2024. Available from: https://developers.google.com/earth-engine/datasets/catalog/MODIS_MOD09GA_006_NDVI [dataset]

[40] 40. Montero D. eemont: A Python package that extends Google Earth Engine. J Open Source Software. 2021;6(62):3168. Available from: https://joss.theoj.org/papers/10.21105/joss.03168.pdf

[41] 41. MapBiomas. MapBiomas Project, 2023, "Collection 8 of the Annual Land Cover and Land Use Maps of Brazil (1985-2022)", https://doi.org/10.58053/MapBiomas/VJIJCL, MapBiomas Data, V1. 2023. Accessed Nov, 2023. Available from: Available from: https://brasil.mapbiomas.org/estatisticas/ [dataset]

[42] 42. Allik M, Ramos D, Agranonik M, Pinto-Júnior EP, Ichihara MY, Barreto ML, et al. Developing a small-area deprivation measure for Brazil: Technical report. National Institute for Health Research; Cidacs/University of Glasgow; 2020. Accessed 30 Jan, 2023. Available from: https://doi.org/10.36399/gla.pubs.215898 https://cidacs.bahia.fiocruz.br/ [dataset]

[43] 43. INMET - National Institute of Meteorology. Banco de dados meteorológicos. Brasília: Instituto Nacional de Meteorologia; 2022. Available from: https://portal.inmet.gov.br/ [dataset]

[44] 44. Fick SE, Hijmans RJ. WorldClim 2: new 1km spatial resolution climate surfaces for global land areas. Int J Climatol. 2017;37(12):4302-15. Available from: https://doi.org/10.1002/joc.5086 [dataset]

[45] 45. WorldClim, 2023. Global climate and weather data. Historical monthly weather data. Accessed 16 Apr, 2023. Available from: Available from: https://www.worldclim.org/data/monthlywth.html [dataset]

[46] 46. Palasio RGS, Bermudi PM, Macedo FLL, Santana LMR, Chiaravalloti-Neto F. Zika, chikungunya and co-occurrence in Brazil: space-time clusters and associated environmental-socioeconomic factors. Sci Rep. 2023;13(1):18026. Available from: https://doi.org/10.1038/s41598-023-42930-4 10.1038/s41598-023-42930-4 PMC10590386 37865641

[47] 47. Lima MAO, Cerqueira HML, Almeida IFB, Lima MM, Cerqueira EM, Alcântara LCJ. Distribuição espacial de dengue, chikungunya e Zika e os determinantes socioeconômicos em um município da Bahia. Rev Ciênc Méd Biol. 2022;20(4):551-9. Available from: https://doi.org/10.9771/cmbio.v20i4.38344

[48] 48. Lippi CA, Stewart-Ibarra AM, Loor ME, Zambrano J, López-Gatell H, Blackburn JK, et al. The social and spatial ecology of dengue presence and burden during an outbreak in Guayaquil, Ecuador, 2012. Int J Environ Res Public Health. 2018;15(4):827. Available from: https://doi.org/10.3390/ijerph15040827 10.3390/ijerph15040827 PMC5923869 29690593

[49] 49. Queiroz ERS, Medronho RA. Spatial analysis of the incidence of Dengue, Zika and Chikungunya and socioeconomic determinants in the city of Rio de Janeiro, Brazil. Epidemiol Infect. 2021;149:e188. Available from: https://doi.org/10.1017/S0950268821001801 10.1017/S0950268821001801 PMC8365848 34338179

[50] 50. IBGE - Instituto Brasileiro de Geografia e Estatística. Pesquisa nacional de saneamento básico 2008 [Internet]. Rio de Janeiro: IBGE ; 2010. Accessed Jan 30, 2023. Available from: Available from: https://biblioteca.ibge.gov.br/visualizacao/livros/liv45351 .pdf

[51] 51. Ruf F, Schroth G. Chocolate forests and monocultures: a historical review of cocoa growing and its conflicting role in tropical deforestation and forest conservation. In: Schroth G, da Fonseca GAB, Harvey CA, Gascon C, Vasconcelos HL, Izac AMN, editors. Agroforestry and Biodiversity Conservation in Tropical Landscapes. Washington, DC: Island Press; 2004. 107-34 p.

[52] 52. Fernandes MM, Fernandes MRM, Garcia JR, Eraldo Matricardi AT, Lima AHS, et al. Land use and land cover changes and carbon stock valuation in the São Francisco river basin, Brazil. Environ. Challenges. 2021;5:100247. Available from: https://doi.org/10.1016/j.envc.2021.100247

[53] 53. Souza JHM, Barros TB, Almeida PP, Vieira SCA, Melo FF, Silva RAA, et al. Dynamics of transmission of urban arbovirus dengue, Zika and Chikungunya in southwestern region of Bahia, Brazil. An Acad Bras Cienc. 2021;93(3):e20200670. Available from: https://doi.org/10.1590/0001-3765202120200670 10.1590/0001-3765202120200670 33681889

[54] 54. Cirilo MVF, Pour SZ, Benedetti VF, Farias JP, Fogaça MMC, Simões RC, et al. Co-circulation of Chikungunya virus, Zika virus, and serotype 1 of Dengue virus in Western Bahia, Brazil. Front Microbiol. 2023;14:1240860. Available from: https://doi.org/10.3389/fmicb.2023.1240860 10.3389/fmicb.2023.1240860 PMC10482036 37680530

[55] 55. Mariano-Neto E, Santos RAS. Changes in the functional diversity of birds due to habitat loss in the Brazil Atlantic Forest. Front For Glob Change. 2023;6:1041268. Available from: https://doi.org/10.3389/ffgc.2023.1041268

[56] 56. Keesing F, Belden LK, Daszak P, Dobson A, Harvell CD, Holt RD, et al. Impacts of biodiversity on the emergence and transmission of infectious diseases. Nature. 2010;468(7324):647-52. Available from: https://doi.org/10.1038/nature09575 10.1038/nature09575 PMC7094913 21124449

[57] 57. Lorenz C, Castro MC, Trindade PMP, Nogueira ML, Lage MO, Quintanilha JA, et al. Predicting Aedes aegypti infestation using landscape and thermal features. Sci Rep. 2020;21688(2020). Available from: https://doi.org/10.1038/s41598-020-78755-8 10.1038/s41598-020-78755-8 PMC7729962 33303912

[58] 58. Vieira CJSP, São Bernardo CS, da Silva DJF, Kubiszeski JR, Barreto ES, Monteiro HAO, Canale GR, et al. Land-use effects on mosquito biodiversity and potential arbovirus emergence in the Southern Amazon, Brazil. Transbound Emerg Dis. 2022;69(4):1770-81. Available from: https://doi.org/10.1111/tbed.14154 10.1111/tbed.14154 33993650

[59] 59. Abdullah NAMH, Dom NC, Salleh SA, Salim H, Precha N. The association between dengue case and climate: A systematic review and meta-analysis. One Health. 2022;15:100452. Available from: https://doi.org/10.1016/j.onehlt.2022.100452 10.1016/j.onehlt.2022.100452 PMC9767811 36561711

[60] 60. Catenacci LS, Ferreira MS, Fernandes D, Padda H, Travassos-da-Rosa ES, Deem SL, et al. Individual, household and environmental factors associated with arboviruses in rural human populations, Brazil. Zoonoses Public Hlth. 2021;68(3):203-12. Available from: https://doi.org/10.1111/zph.12811 10.1111/zph.12811 33538403

[61] 61. Delrieu M, Martinet JP, O'Connor O, Viennet E, Menkes C, Burtet-Sarramegna V, et al. Temperature and transmission of chikungunya, dengue, and Zika viruses: A systematic review of experimental studies on Aedes aegypti and Aedes albopictus. Curr Res Parasitol Vector-Borne Dis. 2023;4:100139. Available from: https://doi.org/10.1016/j.crpvbd.2023.100139. 10.1016/j.crpvbd.2023.100139 PMC10500480 37719233

[62] 62. Iwamura T, Guzman-Holst A, Murray KA. Accelerating invasion potential of disease vector Aedes aegypti under climate change. Nat Commun. 2020;11(1):2130. Available from: https://doi.org/10.1038/s41467-020-16010-4 10.1038/s41467-020-16010-4 PMC7195482 32358588

[63] 63. Li F, Zhou H, Huang DS, Guan P. Global research output and theme trends on climate change and infectious diseases: a retrospective bibliometric and co-word biclustering investigation of papers indexed in PubMed (1999-2018). Int J Environ Res Public Health. 2020;17(14):5228. Available from: https://doi.org/10.3390/ijerph17145228 10.3390/ijerph17145228 PMC7400491 32698499

[64] 64. Kakarla SG, Mopuri R, Mutheneni SR, Bhimala KR, Kumaraswamy S, Kadiri MR, et al. Temperature dependent transmission potential model for chikungunya in India. Sci Total Environ. 2019;647:66-74. Available from: https://doi.org/10.1016/j.scitotenv.2018.07.461 10.1016/j.scitotenv.2018.07.461 30077856

[65] 65. Freitas LP, Schmidt AM, Cossich W, Cruz OG, Carvalho MS. Spatio-temporal modelling of the first Chikungunya epidemic in an intra-urban setting: The role of socioeconomic status, environment and temperature. PLoS Negl Trop Dis. 2021;15(6):e0009537. Available from: https://doi.org/10.1371/journal.pntd.0009537 10.1371/journal.pntd.0009537 PMC8244893 34143771

[66] 66. IPCC. Climate Change 2022: Impacts, Adaptation, and Vulnerability. Contribution of Working Group II to the Sixth Assessment Report of the Intergovernmental Panel on Climate Change. P¨ortner HO, Roberts DC, Tignor M,Poloczanska ES, Mintenbeck K, Alegr´ıa A, et al., editors. Cambridge, UK and New York, NY, USA: Cambridge University Press; 2022. Available from: https://doi.org/10.1017/9781009325844.

[67] 67. Ning X, Xia B, Wang J, Gao R, Ren H. Host-adaptive mutations in Chikungunya virus genome. Virulence. 2024;15(1):2401985. Available from: https://doi.org/10.1080/21505594.2024.2401985 10.1080/21505594.2024.2401985 PMC11404619 39263937

[68] 68. Almeida-Souza PA, Oliveira CH de, Brito LP, Teixeira T de J, Celestino IA, Penha GB, et al. High frequencies of kdr mutation and Chikungunya infection in Aedes aegypti population from Minas Gerais, Brazil. Pathogens. 2024;13(6):457. Available from: https://doi.org/10.3390/pathogens13060457 10.3390/pathogens13060457 PMC11206328 38921757

[69] 69. El-Sayed A, Kamel M. Climatic changes and their role in emergence and re-emergence of diseases. Environ Sci Pollut Res Int. 2020;27(18):22336-52. Available from: https://doi.org/10.1007/s11356-020-08896-w 10.1007/s11356-020-08896-w PMC7187803 32347486

[70] 70. Achee NL, Grieco JP, Vatandoost H, Seixas G, Pinto J, Ching-Ng L, et al. Alternative strategies for mosquito-borne arbovirus control. PLoS Negl Trop Dis. 2019;13(1):e0006822. Available from: https://doi.org/10.1371/journal.pntd.0006822 10.1371/journal.pntd.0006822 PMC6317787 30605475

[71] 71. Wermelinger ED. Interdisciplinaridade na estratégia de controle dos vetores urbanos das arboviroses: uma dimensão necessária para o Brasil. Cad Saúde Pública. 2022;38(1):e00243321. Available from: https://doi.org/10.1590/0102-311X00243321 10.1590/0102-311X00243321 35081204

